# Supplementary figures and images for: Couple oriented counselling improves male partner involvement in sexual and reproductive health of a couple: Evidence from the ANRS PRENAHTEST randomized trial
Source: PLoS One. 2021 Jul 30;16(7):e0255330. doi: 10.1371/journal.pone.0255330 (PMC8323939; doi:10.1371/journal.pone.0255330)

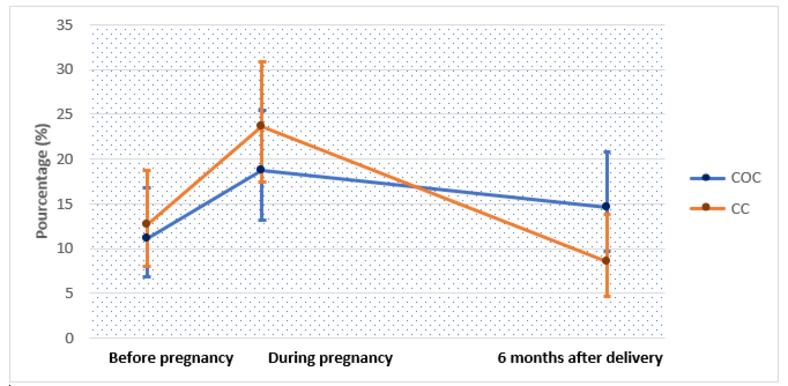

Supplement: S1 Fig — (TIF) [file pone.0255330.s001.tif]

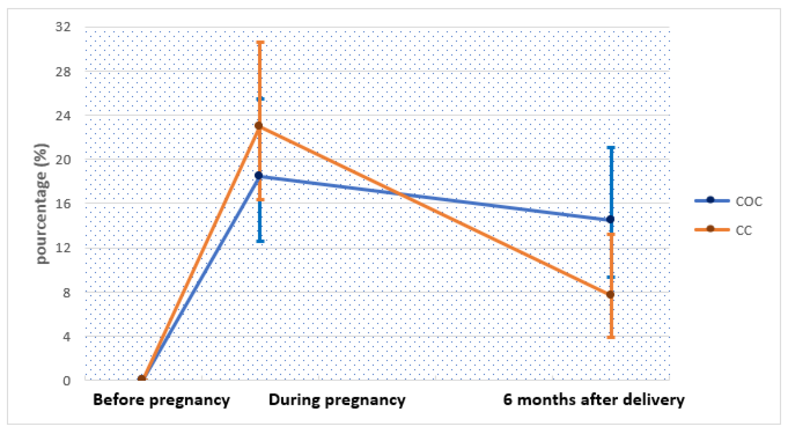

Supplement: S2 Fig — (TIF) [file pone.0255330.s002.tif]
