# Supplementary material for: Couple oriented counselling improves male partner involvement in sexual and reproductive health of a couple: Evidence from the ANRS PRENAHTEST randomized trial
Source: PLoS One. 2021 Jul 30;16(7):e0255330. doi: 10.1371/journal.pone.0255330 (PMC8323939; doi:10.1371/journal.pone.0255330)
Supplement: S1 Table — (PDF) [file pone.0255330.s003.pdf]

<u>S1 Table:</u> Description of the MPI clusters before pregnancy, during pregnancy and six months after delievery obtained from mixed classification, Prenahtest ANRS 12127-12236 Prenahtest, Cameroon, 2009-2011.

**TIME VISIT**	**Before pregnancy**		**During pregnancy**		**Six months after delivery**	
**CLUSTER 1 / 2:** ***Low MPI****	**(N= 422 - %= 88.28)**		**(N = 333 - %= 80.24)**		**(N= 305 - %= 88.15)**	
**Variable labels**	**Characteristic modalities**	**Test - Value**	**Characteristic modalities**	**Test - Value**	**Characteristic modalities**	**Test - Value**
Disclosure of HIV test results within 6 months	No	17.86				
Uptake of HIV test within 6 months	No	17.46				
Discussion about HIV initiated by male partner within the couple	No	3.31				
Accompaniment for the woman in the consultation			No	13.00		
Disclosure of HIV test results since last interview			No	9.65		
Uptake of HIV test result since last interview			No	9.49		
Communication initiated by male partner about condom within the couple			No	5.61		
Discussion about HIV initiated by male partner within the couple			No	4.64		
Disclosure of HIV test results since last interview					No	99.99
Uptake of HIV test result since last interview					No	99.99
Low risk of sexual intercourse					No	1.74
**CLUSTER 2 / 2: High MPI***	(N=56 - %= 11.72)		**(N=82 - %= 19.76)**		(N=41 - %= 11.85)	
**Variable labels**	**Characteristic modalities**	**Test - Value**	**Characteristic modalities**	**Test - Value**	**Characteristic modalities**	**Test - Value**
Disclosure of HIV test results within 6 months	Yes	17.86				
Uptake of HIV test within 6 months	Yes	17.46				
Discussion about HIV initiated by male partner within the couple	Yes	3.31				
Accompaniment for the woman in the consultation			Yes	13.00		
Disclosure of HIV test results since last interview			Yes	9.65		
Uptake of HIV test result since last interview			Yes	9.49		
Communication initiated by male partner about condom within the couple			Yes	5.61		
Disclosure of HIV test results since last interview					Yes	99.99
Uptake of HIV test result since last interview					Yes	99.99
Low risk of sexual intercourse					Yes	1.74

MPI : Male Partner Involvement
