# Supplementary material for: Couple oriented counselling improves male partner involvement in sexual and reproductive health of a couple: Evidence from the ANRS PRENAHTEST randomized trial
Source: PLoS One. 2021 Jul 30;16(7):e0255330. doi: 10.1371/journal.pone.0255330 (PMC8323939; doi:10.1371/journal.pone.0255330)
Supplement: S2 Table — (PDF) [file pone.0255330.s004.pdf]

<u>S2 Table:</u> Description of the MPI clusters from mixed classification during follow-up, Prenahtest ANRS 12127-12236 Prenahtest, Cameroon, 2009-2011.

**MPI Level**	**Before pregnancy**		**During pregnancy**		**After Delivery**	
	**N**	**%**	**N**	**%**	**N**	**%**
Low MPI	**422**	**88.28**	**333**	**80.24**	**305**	**88.15**
CC	207	86.61	166	79.05	155	91.18
COC	215	89.96	167	81.46	150	85.23
High MPI	**56**	**11.72**	**82**	**19.76**	**41**	**11.85**
CC	32	13.39	44	20.95	15	8.82
COC	24	10.04	38	18.54	26	14.77
