# Supplementary material for: Couple oriented counselling improves male partner involvement in sexual and reproductive health of a couple: Evidence from the ANRS PRENAHTEST randomized trial
Source: PLoS One. 2021 Jul 30;16(7):e0255330. doi: 10.1371/journal.pone.0255330 (PMC8323939; doi:10.1371/journal.pone.0255330)
Supplement: S1 Result — (PDF) [file pone.0255330.s005.pdf]

Supplement: **Couple oriented counselling improves male partner involvement in sexual and reproductive health of the couple: evidence from the ANRS 12127/12236 PRENAHTEST Cohort in Cameroon.**

S1 Results: this trend could be influenced by lost to follow-up (permanent or intermittent). To verify this hypothesis, we only represented the evolution of the high MPI for whom participated in all the visits during follow-up (S1 Fig). It turned out that this hypothesis is not verified because we always observe the same trend. Furthermore, we note that before pregnancy (at the time of randomization), we had differences in the proportion of high MPI between the SC and COC groups: even if the difference is not significant, this could constitute a bias in what was observed during the follow-up, since the two groups are not well balanced at randomization according to MPI. To verify this presumption, we only represented the evolution of the high MPI for whom participated in all visits during follow-up and whose partners were not involved before randomization (before participating in the post-test counseling) (S2 Fig). We observe the same trend as Fig.2 and S1 Fig.
